# Tumors Widely Express Hundreds of Embryonic Germline Genes

**DOI:** 10.3390/cancers12123812

**Published:** 2020-12-17

**Authors:** Jan Willem Bruggeman, Naoko Irie, Paul Lodder, Ans M. M. van Pelt, Jan Koster, Geert Hamer

**Affiliations:** 1 Reproductive Biology Laboratory, Center for Reproductive Medicine, Amsterdam Research Institute Reproduction and Development, Amsterdam University Medical Centers, University of Amsterdam, 1105 AZ Amsterdam, The Netherlands; j.bruggeman@amsterdamumc.nl (J.W.B.); paul_lodder@live.nl (P.L.); a.m.vanpelt@amsterdamumc.nl (A.M.M.v.P.); 2 Wellcome Trust/Cancer Research UK Gurdon Institute, University of Cambridge, Tennis Court Road, Cambridge CB2 1QN, UK; ni241@cam.ac.uk; 3 Department of Oncogenomics, Amsterdam University Medical Centers, University of Amsterdam, 1105 AZ Amsterdam, The Netherlands; jankoster@amsterdamumc.nl

**Keywords:** primordial germ cells (PGCs), germline, germ cell cancer genes (GC genes), cancer/testis genes (CT genes), oncogenesis, cancer treatment development, fertility preservation

## Abstract

**Simple Summary:**

Many current cancer treatments also target healthy cells, leading to severe side effects, including infertility. Alternative more cancer specific targets, not expressed in any healthy tissue, would result in fewer side effects. To this aim we identified hundreds of genes expressed by cancer cells that are normally only expressed during the embryonic development of reproductive cells. Because these genes are not expressed in any healthy adult tissue, including the adult testis or ovary, their possible targeting is likely not to have any side effects. Moreover, many of these germ cell genes appear to contribute to typical cancer cell characteristics such as genome instability and metastasis. We indeed found that a relatively high amount of germ cell genes in lung cancer leads to a poor prognosis. We expect that these results will lead to a better understanding of cancer and provide multiple new targets with reduced side effects.

**Abstract:**

We have recently described a class of 756 genes that are widely expressed in cancers, but are normally restricted to adult germ cells, referred to as germ cell cancer genes (GC genes). We hypothesized that carcinogenesis involves the reactivation of biomolecular processes and regulatory mechanisms that, under normal circumstances, are restricted to germline development. This would imply that cancer cells share gene expression profiles with primordial germ cells (PGCs). We therefore compared the transcriptomes of human PGCs (hPGCs) and PGC-like cells (PGCLCs) with 17,382 samples from 54 healthy somatic tissues (GTEx) and 11,003 samples from 33 tumor types (TCGA), and identified 672 GC genes, expanding the known GC gene pool by 387 genes (51%). We found that GC genes are expressed in clusters that are often expressed in multiple tumor types. Moreover, the amount of GC gene expression correlates with poor survival in patients with lung adenocarcinoma. As GC genes specific to the embryonic germline are not expressed in any adult tissue, targeting these in cancer treatment may result in fewer side effects than targeting conventional cancer/testis (CT) or GC genes and may preserve fertility. We anticipate that our extended GC dataset enables improved understanding of tumor development and may provide multiple novel targets for cancer treatment development.

## 1. Introduction

Many genes have been identified that drive the transition from healthy cells into cancer cells. Such oncogenes contribute to the acquisition of cancer-specific hallmarks [1,2], such as uncontrolled cell divisions, angiogenesis, aberrant apoptosis regulation, and telomere maintenance. Targeting these hallmark processes is effectively done by many current cancer therapies. However, because the majority of these processes are also widely used by non-cancerous cells, these therapies often cause severe side effects. To diminish potential side effects in cancer therapy, the identification of oncogenes that are inactive in mature healthy human tissues is paramount to the development of novel diagnostics and therapeutics.

**Figure 1:**
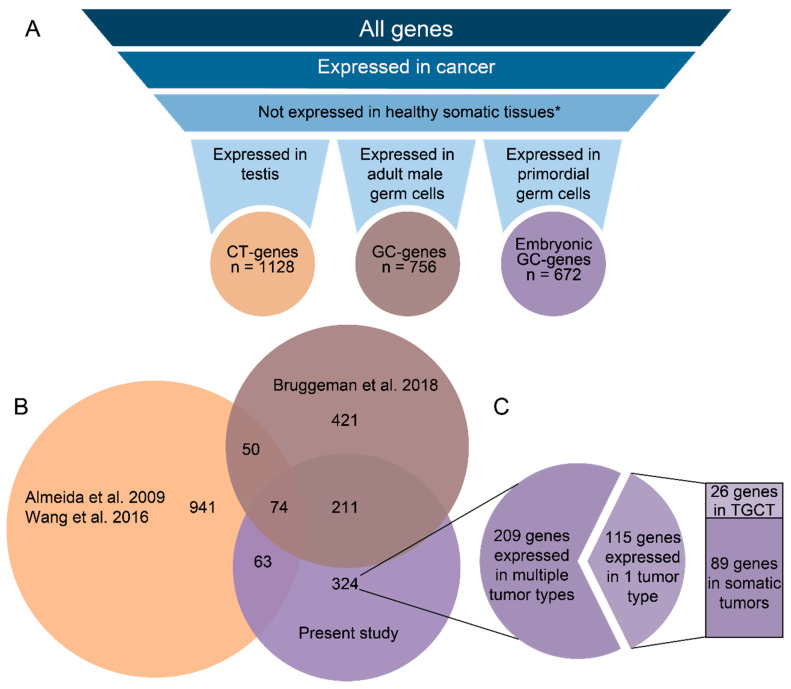
(**A**) Visualization of how cancer/testis (CT) and germline/cancer (GC) genes have been identified. Left: genes included by Wang et al. [6] and Almeida et al. [5] have been based on gene expression in whole testis tissue (CT genes). Middle: genes included by Bruggeman et al. [7] have been based on gene expression in germ cells (GC genes). Right: Genes included in the present analysis have been included based on gene expression in human primordial germ cells (GC genes). * = testis and ovary were excluded as they are not considered somatic. (**B**) Approximately half of the embryonic GC genes have not yet been described before as GC genes or CT genes. Venn diagram comparing the present analysis of human GC genes expressed in primordial germ cells (red) to earlier identified GC genes expressed in adult germ cells [8] and cancer/testis (CT) genes [5,6] (Data S1A). (**C**) The majority of newly identified embryonic GC genes are expressed in multiple tumor types. From the 115 genes expressed in only one tumor type, 26 are expressed in testicular germ cell tumors (TGCTs), showing that the majority of GC genes are expressed in tumors that originate from somatic tissues.

One group of genes that has been studied to this end are cancer/testis (CT) genes [3,4]. CT genes have been identified by selecting genes that are highly expressed in testis tissue and cancer, and expressed in a limited number of healthy somatic tissues. This approach has resulted in the identification of 1128 CT genes to date (Figure 1A) [5,6]. However, CT genes include the genes expressed in somatic cells in the testis, precluding the detection of true germ-cell-specific genes. By using the transcriptome of isolated adult male germ cells, we have recently identified 756 true germ-cell-specific genes expressed in cancer, termed germline/cancer genes (GC genes), of which 630 (83%) were newly identified (Figure 1A,B) [7].

Cancer and germ cells share many biological properties, such as “endless/immortal” propagation and developmental potential. Indeed, we found that GC genes are involved in processes that can drive and maintain tumor development [7]. This supports the hypothesis that cancer initiation and early development (carcinogenesis) involves biomolecular processes and regulatory mechanisms that are usually restricted to germ cell development [4,7,9,10,11,12,13,14]. To investigate this, we sought the oncogenic potential of molecular regulation for the primordial germ cells (PGCs), the embryonic precursors of the adult germ cells, which has not been investigated. Human PGCs are specified in the week 2 embryo by expression of the transcription factor SOX17 [15], resulting in global hypomethylation and latent pluripotency. Moreover, the process can be recapitulated in vitro by using human pluripotent stem cells [15,16,17]. Specified PGCs in embryos multiply extensively and migrate from their site of origin in the proximal epiblast, through the developing hindgut, to the gonadal ridges during week 3 to 5 of human development [18]. As such, the physiology of PGCs includes processes that also have been proposed as hallmarks of cancer, including continuous replicative potential via telomere lengthening [19], deregulating cellular energetics [20], and invasive potential and metastasis [21]. Furthermore, DNA hypomethylation, in itself a characteristic feature of PGCs [22], is also a proposed consequence of germ-cell-specific gene activity in tumors [23]. As these processes favor development and survival of the cancer cell, investigating genes specific to PGCs and cancer has great potential for tumor biology. When a therapeutic target is unique to cancer and adult germ cells, side effects of targeting such gene products during cancer therapy would be limited to (temporary) infertility. However, targeting genes specific to PGCs may not even result in any side effects because these gene products are absent from the adult germ cells.

## 2. Results

To assemble an inventory of genes of interest, we explored the gene expression of tumor data from the TCGA [24], normal tissue expression from GTEx [25] and primordial germ cell data from Irie et al. 2015 [15]. By applying similarly strict inclusion criteria as in our previous study [7] (Figure S1), here we identify 672 genes that are expressed in primordial germ cells (i.e., either human PGCs derived from week 5.5–8.5 embryos and/or in vitro derived PGCLCs representing week 2.5–3.0 of development) and a wide variety of tumor types, while being virtually undetectable from the GTEx database of healthy somatic tissues (Figure 1A, Data S2A). As they are expressed in the embryonic germline, we will refer to these genes as “embryonic” GC genes; 348 genes (51%) are also expressed in mature germ cells or testis tissue and have been identified as GC or CT genes before (Figure 1B, Data S2A). We thus expand the known CT/GC gene pool [5,6,7] with 324 new genes that are restricted to the germline and cancer (Figure 1C). We have visualized how custom inclusion criteria affect the results and their overlap with other studies in a Web-based application, available from http://venn.lodder.dev. A Gene Ontology (GO) analysis suggests that the 672 genes expressed in PGCs and cancer cells play roles in unique processes, including the meiotic cell cycle, nucleic acid metabolic processes, nuclear division, strand displacement, gene regulation, and stem cell population maintenance (Table S1, Data S2B).

### 2.1. GC Genes Can be Classified into Groups Based on Similar Expression Profiles in Cancer

**Figure 2:**
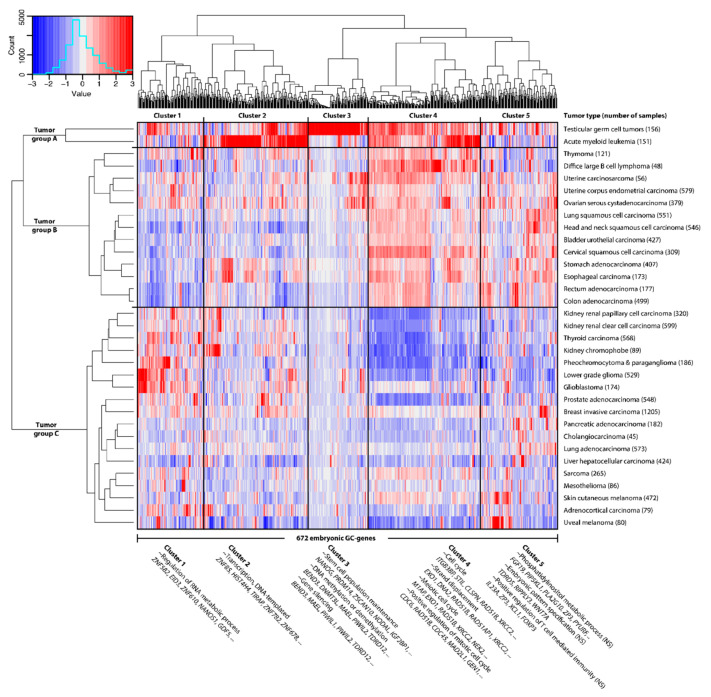
Hundreds of primordial germ-cell-specific genes are widely expressed in tumors—shown here as hierarchal clustering of the average expression per tumor group (Euclidean distance, ward linkage). These 672 embryonic germline/cancer (GC) genes expressed in primordial germ cells divide tumors into two groups, mainly based on embryonic GC gene cluster 4, which contains genes involved in the mitotic and meiotic cell cycles (Table S1). Gene expression levels in tumors are indicated by a Z-score dependent color, where blue and red represent low and high expression respectively. These Z-scores are based on expression values (Data S2A, columns DN up to and including ET). Representative and significantly enriched Gene Ontology (GO) terms and several associated genes are shown. Cluster 5 contains no significantly (NS) enriched GO terms.

To investigate whether subgroups of embryonic GC genes differ per tumor type, we performed an unsupervised hierarchal clustering of the 672 embryonic GC genes and the 33 tumor types. This resulted in five subgroups of genes that show similar expression within tumors, and three subgroups of tumors that show similar embryonic GC gene expression profiles (Figure 2). GC genes in cluster 1 appeared to be particularly expressed in lower grade glioma and glioblastoma, and pheochromocytoma and paraganglioma, and that cluster seems to contain genes that regulate RNA metabolic processes (Table S1 and Data S3A). Gene cluster 2 mostly characterizes tumor group A, because it contains many genes that are expressed in acute myeloid leukemia. These genes are associated with DNA-templated transcription (Table S1 and Data S3B). The GC genes in cluster 3 appeared to include the majority of genes that are highly expressed in testicular germ cell tumors and are not expressed in any other tumor type. These genes are mainly responsible for stem cell population maintenance and epigenetic changes (Table S1 and Data S3C). Gene cluster 4 appears to be the main determinant that separates tumor group C from groups A and B. Characterization of gene cluster 4 by GO analysis showed that these GC genes are responsible for many processes related to the meiotic and mitotic cell cycle (Table S1 and Data S3D). A GO analysis of gene cluster 5 showed no significantly upregulated processes (Data S3E).

In order to support the validity of the GC genes and the described clusters, we performed a gene set enrichment analysis (GSEA), in which all 672 embryonic GC genes or the five clusters were defined as gene sets. This analysis showed that embryonic GC genes are highly enriched among genes that are differentially expressed between cancer and normal tissues in our dataset (enrichment score 0.83, *p* = 0.000). This also independently holds for each of the five clusters (Figure S2 and Data S3F).

### 2.2. Embryonic GC Genes are Often Expressed in Multiple Tumor Types

Besides these gene clusters, the set of 672 embryonic GC genes expressed in PGCs contains several subgroups of interest, such as GC genes that are expressed in more than one type of cancer. In the heat map (Figure 2) we observe that most genes are expressed in multiple tumor types, even though the selection criteria allow for the inclusion of genes expressed in only one tumor type. Whereas 35% of embryonic GC genes are expressed in only one tumor type, 138 embryonic GC genes (21%) are expressed in at least half (i.e., 17 or more) of all investigated tumor types (Data S4A). Due to their expression profiles across tumors of different origins, we hypothesize that these GC genes contribute to hallmarks of cancer and that tumors may be dependent on expression of a large subset of GC genes. Characterization by a GO analysis revealed that genes expressed in 17 or more tumor types are responsible for proliferation (i.e., cell cycle processes and positive regulation of mitosis) and genome instability (i.e., chromosome segregation; DNA repair and response to radiation) (Table S1 and Data S4B). Oppositely to this group, some genes have been included because they are expressed in only one tumor type. This particularly holds for genes expressed in testicular germ cell tumors (TGCTs), as they may resemble and originate from (primordial) germ cells. Eighty embryonic GC genes (11%) have been included in our selection because of high expression in TGCTs (Data S5A), of which 70 are in gene cluster 3. Gene ontology analysis showed this subset of genes is involved in cellular aromatic compound metabolic processes, reproductive processes, DNA (de)methylation, and stem cell population maintenance (Data S5B). The other 592 embryonic GC genes (89%) are expressed in at least one tumor type of somatic origin.

### 2.3. A GC Gene Signature Score to Rate Shared Properties between Cancer and the Germline

**Figure 3:**
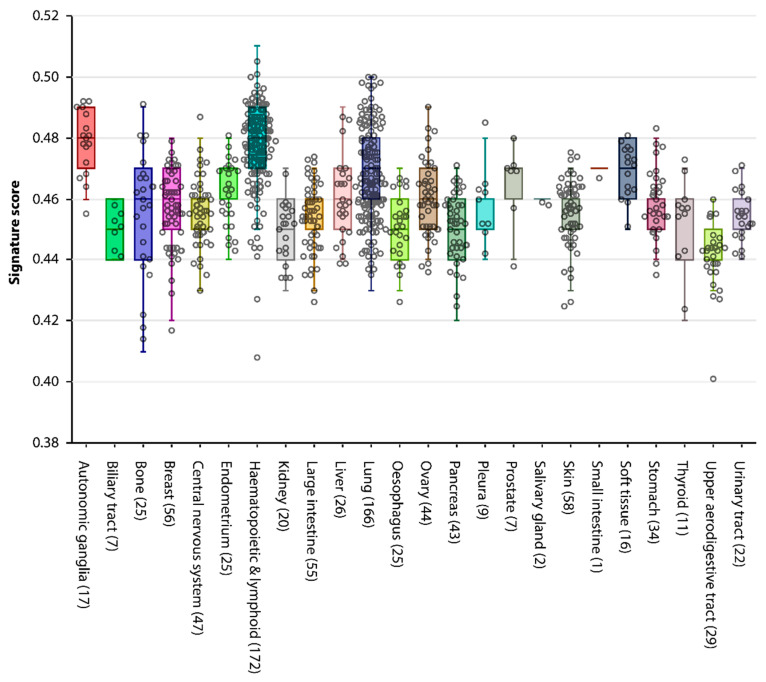
GC signature score in 917 cancer cell lines in the Cancer Cell Line Encyclopedia [27], based on all 1143 known GC genes. Every dot represents one cancer cell line. The signature score is the average percentile of ranked gene expression in each cell line, and may be used as a measure for a cancer cell line’s similarity to the germline.

In the heat map (Figure 2) we observe that some tumors contain many more GC genes than others, ranging from 84 in ovarian serous cystadenocarcinoma and head/neck squamous cell carcinoma to 360 in skin cutaneous melanoma (Data S6A). A tumor’s similarity to the germline thus differs vastly between tumors. In order to quantify this resemblance, we have combined our 672 embryonic GC genes with the previously published 756 GC genes expressed in adult male germ cells [7] (total *n* = 1143, Data S7A), and used the R2 bioinformatics platform [26] to obtain a signature score for each of the 917 publicly available cancer cell lines in the Cancer Cell Line Encyclopedia [27] (Figure 3). This score represents the average percentile of the GC genes expression ranks within a particular cell line, which may be used as a measure of germ cell resemblance. As somatic and non-somatic genes are likely to affect each other’s expression in a tumor, we also used R2 to identify key genes that are not necessarily in our dataset but correlate with the expression of GC genes. We identified 223 genes whose individual expression positively correlates (R > 0.5 and *p* < 0.05) with the GC signature scores (Data S7B) and 277 genes that negatively correlate (R < −0.5 and *p* < 0.05) with the GC signature scores (Data S7C). Interestingly, only 52 of the 223 genes (23%) that positively correlate with the GC signature are GC genes, suggesting that genes leading to activation of the germline program in developing cancer cells may not be expressed in, or exclusive to, the germline.

### 2.4. Expression of GC Genes is Linked to Increased Mortality in Lung Adenocarcinoma

**Figure 4:**
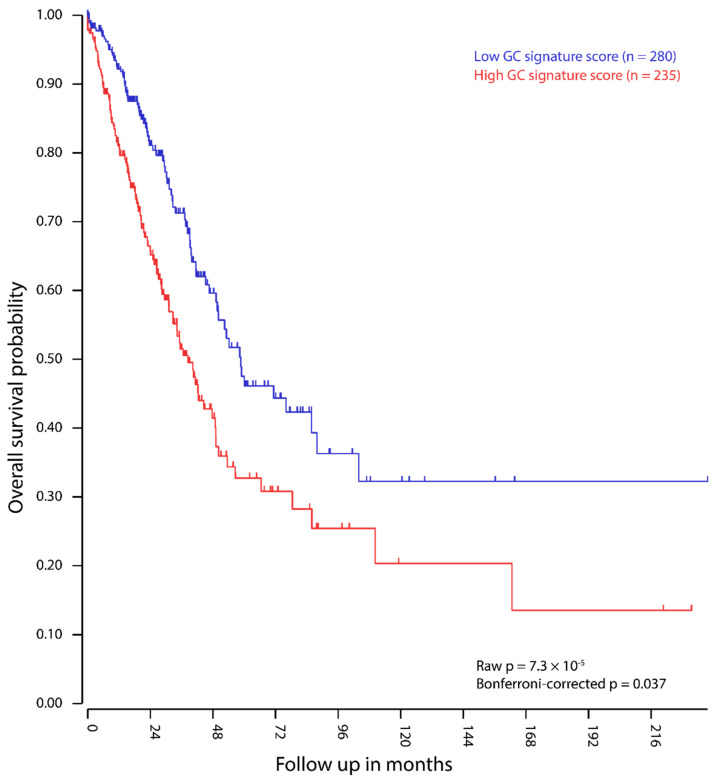
Kaplan–Meier curve of 515 lung adenocarcinoma patients, divided in two groups based on GC gene signature scores of their tumors. Figure derived from the bioinformatics platform R2’s Kaplan Meier Scanner.

As the lung cancer group contains sufficient samples (*n* = 166) and a large variability of GC gene expression between cell lines (Figure 3), we used the most prevalent subtype, lung adenocarcinoma (LUAD), as a model to test whether the expression of GC genes in this tumor-type may influence patient survival. While the decision for LUAD is based on cell line data from the CCLE, the TCGA database contains patient survival data. Using the R2 bioinformatics platform, each of the 515 patient-derived LUAD samples in the TCGA database was attributed a GC signature score based only on the 422 GC genes that are expressed in LUAD. Survival data shows that a high GC gene signature score correlates with increased mortality in LUAD patients (Figure 4, *p* < 0.001).

### 2.5. Highly PGC-Specific Genes Promote Epigenetic Alterations

**Figure 5:**
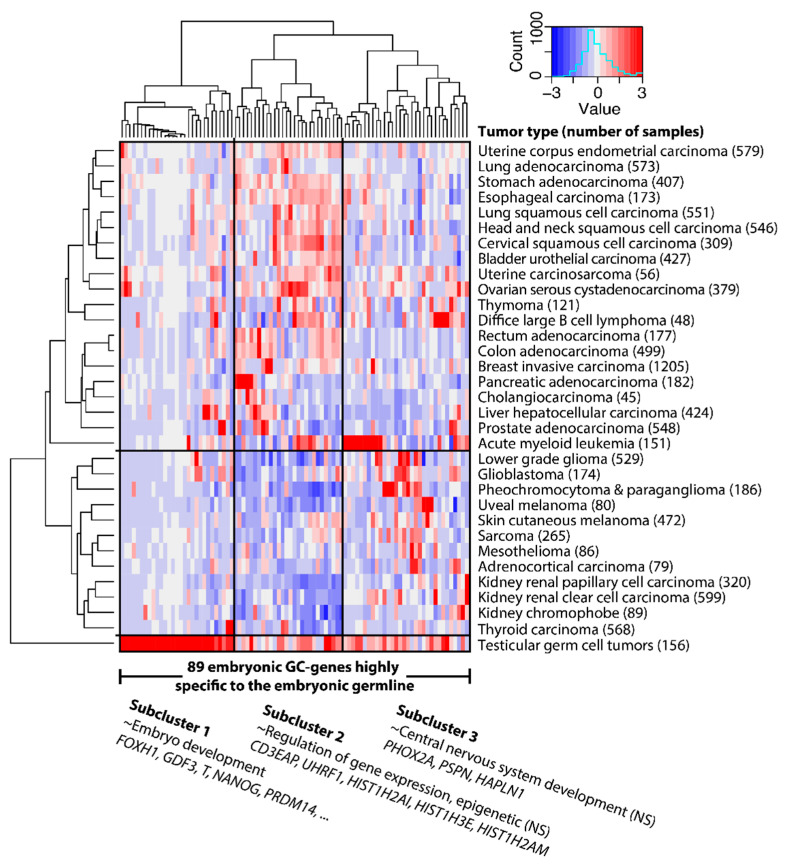
A subset of 89 embryonic GC genes highly specific to the embryonic germline are not only expressed in germ cell tumors, but also in many tumors that originate from tissues of somatic origin. Representative GO terms and some associated genes are known. GO terms are not significantly (NS) enriched, possibly due to the small sample sizes of subclusters. Clusters in Figure 2A and subclusters in Figure 5 were derived independently.

We next determined which embryonic GC genes are only expressed in embryonic stem cells (ESCs) and PGCs, and not in other cells of the germline. We excluded GC genes that show expression in ovary or testis tissue in the GTEx database [25] or adult male germ cells [8], and required a lower expression level in somatic gonadal tissues that surround the PGCs in situ, compared to PGCs [15]. This analysis yielded 89 embryonic GC genes that are highly specific to the embryonic germline and cancer (Figure 5, Data S8A). GO analysis shows that these embryonic GC genes are involved in the regulation of epigenetic gene expression (Table S1, Data S8B). Notably, 21 of these 89 embryonic GC genes are only expressed in TGCT and not in tumors of somatic lineage.

### 2.6. Cell Surface Molecules

As diagnostic and therapeutic targets on the cell surface are more accessible, a final subgroup of interest is genes that encode surface proteins. We therefore used the PANTHER 10.0 classification system to analyze which embryonic GC genes encode cell surface proteins. We identified thirteen of the 672 embryonic GC genes (*ULBP3*, *GP6SPA17*, *CCR4*, *HMMR*, *GP1BA*, *KCNH5*, *UMODL1*, *WNT7A*, *NAT1*, *HYAL4*, *CRLF2*, *TNFSF4*) that are predicted to encode proteins present on the cell surface (Data S9).

### 2.7. Protein Expression

As RNA expression does not necessarily reflect protein expression, we compared our results to data from the Human Protein Atlas (HPA) [28], which contained protein expression data for 374 embryonic GC genes (56%). By not allowing protein expression in any non-cancerous tissue other than ovary and seminiferous tubules of the testis, we identified 37 putative embryonic GC proteins (Data S2A, column L). We continued to use the HPA’s pathology data, containing 20 tumor types, to validate which GC proteins are expressed in tumors, and found that 429 embryonic GC proteins, out of 496 available in the HPA, are expressed in at least one tumor type (Data S2A, column M). Moreover, 284 of these embryonic GC proteins are expressed in at least half of the 20 HPA tumor types.

In addition to the HPA, we used three downloadable mass spectrometry datasets from the Clinical Proteomic Tumor Analysis Consortium (CPTAC) that contain proteomics data for tumor types and matched normal tissues. For LUAD [29], 111 of 206 available embryonic GC proteins (54%) showed higher median protein expression in tumors compared to normal tissues (Data S2A, column N). For colon adenocarcinoma [30], 53 of 90 available embryonic GC proteins (59%) showed higher expression in tumors compared to normal tissues (Data S2A, column O). For uterine corpus endometrial carcinoma [31], 141 of 205 of available embryonic GC proteins (69 %) showed higher median protein expression in tumors compared to normal tissues (Data S2A, column P). In total, protein expression data from either CPTAC or HPA were available for 444 embryonic GC genes, of which 201 could be validated at the protein level (Data S2A, column Q). A GO analysis revealed that these 201 embryonic GC proteins are responsible for chromosome segregation, cell cycle checkpoints, DNA repair, and the meiotic and mitotic cell cycles (Table S1 and Data S12), similarly to gene cluster 4. Indeed, 49% of the embryonic GC genes in gene cluster 4 are among those validated at the protein level. The GC genes that remain unvalidated may be overexpressed in other tumor types than those three, for which the proteome was not available from the CPTAC.

### 2.8. Combination of Subgroups

Finally, we searched for embryonic GC genes that are present in multiple subgroups of interest, those being genes with (i) high embryonic germline specificity, (ii) cell surface expression, and (iii) validation of protein expression according to either HPA or CPTAC data. We found that *NAT1* occurs in all subgroups of interest, and that *HYAL4* encodes a cell surface protein and is highly specific to the embryonic germline. Another 20 embryonic GC proteins were validated at the protein level and in at least one other subgroup of interest (Table S2).

## 3. Discussion

We identified 672 novel germ cell cancer genes (GC genes) that are normally expressed in human primordial germ cells but are ectopically expressed in a wide variety of tumors, most of which are tumors of somatic origin. In addition to existing GC and cancer testis antigens (CT genes), this expansion of the GC gene group is of particular interest to the development of anticancer therapies, as they are not expressed in any healthy adult tissue. Of particular interest are 89 embryonic GC genes that are not expressed in adult germ cells, whole testis tissue, or embryonic somatic gonadal tissue. These genes appear to be involved in the epigenetic regulation of gene expression and gene silencing, which is a key feature of both PGCs and carcinogenesis. As expression of these genes is usually restricted to PGCs, and is thus absent in somatic tissues and adult germ cells, targeting the genes of this subset of GC genes could lead to fewer side effects than existing therapies.

**Figure 6:**
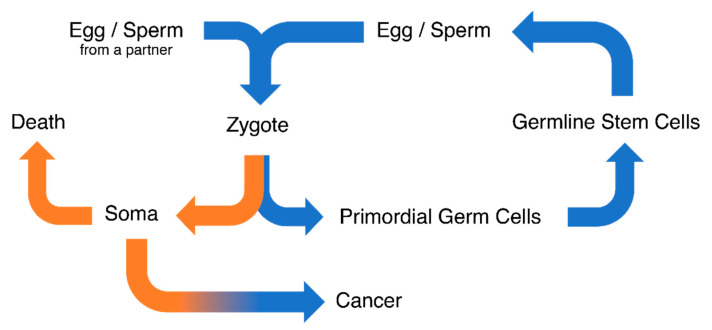
The soma-to-germline oncogenic model: processes related to the human life cycle and cancer development are closely related. Orange: somatic properties. Blue: germline properties.

The expression of testis [5,6,32,33,34,35,36] and germ-cell-specific [4,7,37] genes in tumors has been widely studied. However, gene expression of the embryonic germline had not yet been systematically compared to cancer, despite being suggested in two key publications that sparked CT gene research [3,4]. We show herein that the similarities between cancer and the embryonic germline are widespread and include processes that favor survival of the cancer cell. This finding further supports the “soma-to-germline” oncogenic model, in which the upregulation of germline-specific genes promotes cancer cell development and survival through the acquisition of germ cell-like properties [9,10,11,12,13] (Figure 6). Driven by epigenetic changes, these properties are normally strictly isolated and controlled within the germline, but may allow cancer cells to prioritize their own survival over the survival of the soma. As a consequence, subsequently acquired (pseudo)meiotic functions may help the cancer cells to disturb normal cell cycle regulation and DNA repair mechanisms in order to evade checkpoints and apoptosis [10]. Indeed, and consistently with previous reports on CT genes [38], the expression of the meiosis specific gene *HORMAD1* [39,40], the MAGE-A gene family [41], and the sperm specific cytochrome c oxidase *COX6B2* [42,43], we found that a high GC signature score correlates with poor prognosis in lung adenocarcinoma.

Despite strict criteria for the inclusion of genes based on RNA expression, some genes may have been falsely included or excluded. Firstly, we show that many embryonic GC genes are involved in epigenetic alterations, a well-known mediator of oncogenesis [44], potentially leading to the downregulation of some tumor suppressor genes. As we selected genes based on elevated expression in tumors, downregulated (e.g., tumor suppressor) genes will have escaped our selection, even though their downregulation may be specific to cancer and the germline. On the other hand, we may also have falsely included some genes, specifically those only expressed in somatic cells under specific conditions. This may, for instance, be the case for genes related to mitosis, as hPGCs divide mitotically before initiating meiosis in females around week 10 [45]. Mitosis-associated mRNAs may therefore be relatively overexpressed in hPGCs compared to healthy somatic tissues that do not divide as rapidly. Our GO analyses show that mitosis-related processes are only enriched in cluster 4, which is the main determinant between tumor groups A/B and C. A third reason for the false inclusion or exclusion of genes is the difficulty of detecting genes unique to cell types that are heterogeneous within one tissue, or expressed in other tissues than those assessed in GTEx. For example, we expect genes that are expressed in stem cells and not in differentiated tissues to be lowly expressed in the GTEx database, and thus fall below the level of exclusion. Another example is “whole blood”, which is one of the tissues in the GTEx database that contains many different cell types in varying numbers whose distinctions are not appreciated by our analysis. This could mean that the large number of GC genes that is only expressed in acute myeloid leukemia, and not in any other tumor type, is not specific to AML but might also be physiologically expressed in some myeloid cells. Nevertheless, we have chosen not to exclude these genes from our analysis because they may still be relevant to the treatment development of acute myeloid leukemia, similarly to why we have not excluded 83 GC genes that have only been included due to their expression in TGCTs. Lastly, some genes with low mRNA expression show high protein expression or vice versa [46]. For example, SUV39H2 (also known as KTM1B) is expressed in nearly all tumor types. SUV39H2 is known to negatively regulate gene expression in germ cells [47]. More specifically, it is involved in cell cycle regulation, transcriptional repression, and the regulation of telomere length [48,49]. Deletion of this gene allows for partial elongation of telomeres, thereby aiding tumor growth, suggesting that the presence of SUV39H2 is unlikely to promote tumor growth. This paradox may be explained by posttranscriptional regulation of gene expression, which leads to the accumulation of RNA and the absence of protein. Such posttranscriptional regulation, which also physiologically occurs in germ cell development [8], may have led to the false inclusion of other genes as well. As the present study is based on large datasets comprised of RNA data [8,15,24,25], this remains a notable point of caution. We have attempted to validate these genes on the protein level using the HPA [28] as a validation tool and found 37 putative GC proteins. Despite the increasing reliability of the HPA, several drawbacks remain. Most notably, the antibodies are affinity purified and may thus not be selective for the antigen. For example, NANOG is not expressed in testis tissue according to the HPA, but a study we collaborated in has shown otherwise [50]. We have also used the HPA for pathology data and three CPTAC proteomics datasets to validate the expression of embryonic GC proteins, but protein-level data were not yet available for many embryonic GC genes. Therefore, future research that further explores the therapeutic relevance of individual GC genes should also investigate the GC genes’ encoded proteins or the phenotypic effects of GC gene expression.

As human PGCs differentiation includes dynamic events such as migration and global epigenetic resetting [21], the stage at which they are isolated influences the results. The PGCs used in our analysis were from human embryos that were 5.5–8.5 weeks old, which are similar to mouse PGCs around embryonic day 13 [15,51,52]. Gonadal differentiation is already initiated at this stage [21,45,52], allowing for the attribution of meiosis-related gene activity (gene cluster 4) to the initiation of meiosis in female germ cells. While we hypothesize that PGC migration and cancer metastasis share features, genes that drive this process were probably already downregulated in post-migratory PGCs used in this analysis. It is also unlikely to be able to observe processes related to metastasis in GC genes expressed in PGCLCs, as they represent pre-migratory PGCs in week 2–3. While this might be due to the technical difficulty of isolating migrating PGCs, another challenge in “catching” migration-related processes in our selection is that the tumor dataset only contains primary tumor samples [24]. This means that the RNAs for these genes must already be present before possible metastasis in order to be included. This could indicate that the tumors that express these genes are at an elevated risk of metastasizing. Future research into the migratory potential of (early) germline cells could elucidate to what extent this is the case. In addition, further research into the expression of GC genes in metastatic tumors may yield more putative therapeutic targets that are involved in processes that allow for metastasis.

As human PGCs remain hypomethylated until week 16 [53], it is possible that many genes are randomly expressed in PGCs, which is a feature shared by cancer cells. While we have attempted to achieve tumor specificity through our strict inclusion criteria, a tumor’s true dependency on these genes remains questionable. Even though we have utilized GO analyses to show that embryonic GC genes may be involved in cancer, and that this mechanism of action is plausible, some genes may have been included due to random activation as a consequence of global DNA hypomethylation. As a tumor’s dependency on a potential therapeutic target can be a requirement for the success of a certain therapy [14], this will have to be elucidated at the protein level for every individual gene level.

## 4. Materials and Methods

### 4.1. Datasets

In order to be able to make a selection, we merged several publicly accessible RNA expression datasets (transcriptomes) into one file (Data S6B). In this process, we have used the transcriptomes of hPGCs of week 5.5–8.5 of human embryonic development and PGCLCs representing PGCs in week 2–3 of human embryonic development [15] as base files. We then matched each gene to the expression data from the following transcriptomes in one file (Data S6B):

- The transcriptome of ESCs cultured in conventional media [15].
- The transcriptome of embryonic somatic tissue, which surrounds the hPGCs in situ [15].
- The transcriptome of adult male germ cells in various stages of spermatogenesis [8].
- The Genotype Tissue Expression (GTEx) project, containing 17,382 samples from 54 healthy (i.e., non-cancerous) tissues (Data S6C) [25].
- The Cancer Genome Atlas (TCGA) project, which aims to sequence untreated tumor samples of high biopsy quality, containing 11,003 samples from 33 tumor types (Data S6A) [24].

### 4.2. Selection of Genes

As we compared gene expression levels from multiple sources with distinct distributions, we could not simply compare those values between datasets. Thus, we determined a cut-off for each dataset to include or exclude genes (Figure S1). To be sure that genes are expressed highly in hPGCs, the minimum expression of each gene in any stage (week 5.5–8.5) was compared between female and male hPGCs, after which the maximum value of the two was used to determine arbitrary inclusion criteria. For PGCLCs, inclusion criteria were based on the averages of two samples. Genes showing hPGC expression > 0.72 (33th percentile) or PGCLC expression > 0.50 (33th percentile) were considered expressed (Figure S1A). Additional inclusion criteria were applied to the maximum RNA expression levels per gene in all tissue/tumor types. Namely, genes that are not expressed in any normal tissue (GTEx < 3.0, Figure S1B) and are expressed in at least one tumor type (TCGA > 2.3, Figure S1C) have been included. The values represent the average normalized log_2_(reads per million) in a varying number of patient samples (Data S1B,C). These criteria include genes in the following percentiles: 74% for hPGCs and PGCs, 13% for normal tissues, and 89% for cancer. As the inclusion criteria are arbitrary, we have developed a web-based application that allows anyone to manually change the inclusion criteria and observe how this affects the results: http://r2platform.com/gc_genes/.

### 4.3. Data Analysis

The TCGA (tumor) and GTEx (normal tissues) transcriptome data were downloaded as “processed” matrices, like they are also used in their respective web-resources [8,15,24,25]. The data processing steps are described at https://docs.gdc.cancer.gov/Data/Bioinformatics_Pipelines/Expression_mRNA_Pipeline/#mrna-quantification-command-line-parameters and https://gtexportal.org/home/documentationPage#staticTextAnalysisMethods respectively. The expression estimations used were transcripts per million (TPM) for data from the GTEx; FPKM-UQ for TCGA; and DEseq for data from Irie et al. [15]. All expression values were converted to a log_2_-scale in order to aid the interpretation of fold changes. Since we applied extremes filtering independently on a per data type fashion, different normalization schemes do not pose a problem. Genes that had no expression data available from the GTEx and/or TCGA project were excluded (*n* = 1728, Data S6D), leaving 15,992 genes for our analysis (Data S6B). From the GTEx database, we excluded 2 transformed cell lines. As adult germ cells are present in testis and ovary tissue, we excluded “testis” and “ovary” as well in order to allow for the inclusion of previously identified CT and/or GC genes. The RNA expression levels of both PGC types (hPGCs and PGCLCs) from somatic gonadal tissue and ESCs were corrected for gene length (expression/length*1000) to reduce the number of false positives.

Gene Ontology (GO) analysis was performed with DAVID Bioinformatics Resources [54] v6.8. Gene set enrichment analysis was performed with MSigDB v7.2 [55]. Cellular component analysis was performed by using the Panther 10.0 classification system [56]. Genes associated with the cell surface were those attributed to GO term 9986. Data visualization was done in R2 [26] and the JavaScript library D3. Protein expression was evaluated using the Human Protein Atlas (HPA) [28], available from http://www.proteinatlas.org. For healthy somatic tissues, only proteins categorized as “not detected” in all tissues except the seminiferous tubules and ovaries were classified as “validated”. GC genes were validated at the protein level using publicly available HPA and CPTAC data. Validation criteria for the HPA data were: (i) no protein detection in any healthy somatic tissue and (ii) protein detection in at least one tumor type, where the protein was detected in at least 50% of investigated tumor samples (maximum 12). GC genes were additionally validated at the protein level using data from the National Cancer Institute Clinical Proteomic Tumor Analysis Consortium (CPTAC), for which we specifically used datasets on lung adenocarcinoma [29], colon adenocarcinoma [30], and uterine corpus endometrial carcinoma [31]. GC genes were considered validated at the protein level when the median protein expression in tumor tissues exceeded the median protein expression in matched normal tissues in at least one of the three used CPTAC datasets. CPTAC data were acquired via LinkedOmics [57]. The GC signature scores were attributed using the “sample ranked geneset scores” function in R2, which takes a list of genes and ranks these genes based on expression in a provided set of samples, such as each of the 917 cell lines in the CCLE [27] or each of 515 lung adenocarcinoma tumor samples in the TCGA dataset [24]. The signature score is the average percentile of these ranks, and may thus be used as a measure for a cancer cell line’s similarity to the germline.

## 5. Conclusions

In addition to the previously identified CT and adult GC genes, we here identify 672 genes that are expressed in PGCs and cancer, of which 48% have not been identified as CT or GC genes before. Many of these genes are expressed in multiple tumor types. As these genes are highly specific to tumors, and absent in adult germ cells and somatic tissues, targeting of their gene products is expected to lead to very limited side effects in cancer therapy. We therefore anticipate that this data will not only lead to a better understanding of tumor biology, but also to the development of improved diagnostics and treatment options.

## Supplementary Materials

The following are available online at https://www.mdpi.com/2072-6694/12/12/3812/s1. Figure S1: Selection of genes based on the expression in three datasets. Table S1: Summary of Gene Ontology (GO) analysis of GC genes expressed in primordial germ cells. Table S2: GC genes that are expressed in PGCs and fall into multiple subgroups of interest for further evaluation. Data S1–S12: Excel file containing all supplementary data (legend on first tab).
