# Supplementary material for: Tumors Widely Express Hundreds of Embryonic Germline Genes
Source: Cancers (Basel). 2020 Dec 17;12(12):3812. doi: 10.3390/cancers12123812 (PMC7766889; doi:10.3390/cancers12123812)
Supplement: Supplementary file 1 [file cancers-12-03812-s001.zip › cancers-1033232-XML suppl/cancers-1033232-XML suppl tables.pdf]

cancers

MDPI

Supplementary Tables

Tumors Widely Express Hundreds of Embryonic Germline Genes

Jan Willem Bruggeman, Naoko Irie, Paul Lodder, Ans M. M van Pelt, Jan Koster and Geert Hamer

Table S1. Summary of gene ontology (GO) analysis of GC-genes expressed in primordial germ cells.

Set	Description	Enrichment
All GC-genes expressed in primordial germ cells (n = 672), SD 2B	Nucleic acid metabolic process	9.00
	Nuclear division	7.89
	DNA metabolic process	6.91
	Strand displacement	5.43
	Meiotic cell cycle	4.79
	DNA conformation change	3.27
	Strand displacement	3.24
	Blastocyst development	2.93
	Cell cycle checkpoint	2.78
	Regulation of gene expression, epigenetic	2.58
	Cell cycle phase transition	2.58
	Stem cell population maintenance	2.33
	DNA alkylation	2.31
	Regulation of meiotic cell cycle	2.10
	Centrosome organization	2.09
	Regulation of meiotic cell cycle	2.05
	DNA-dependent DNA replication maintenance of fidelity	1.88
	DNA geometric change	1.85
	Mitotic spindle organization	1.72
	Regulation of DNA recombination	1.52
	Macromolecule methylation	1.36
GC cluster 1 (n = 105), SD 3A	Regulation of RNA metabolic process	1.64
GC cluster 2 (n = 167), SD 3B	Transcription, DNA-templated	6.90
GC cluster 3 (n = 97), SD 3C	Stem cell population maintenance	6.24
	Regulation of cellular macromolecule biosynthetic process	4.56
	Reproductive process	4.21
	Negative regulation of gene expression	2.81
	DNA methylation or demethylation	2.28
	Blastocyst formation	1.71
	Multi-multicellular organism process	1.56
	Gene silencing	1.32
GC cluster 4 (n = 180), SD 3D	Cell cycle	29.74
	Cell cycle	24.73
	DNA conformation change	7.40
	Cell cycle phase transition	7.02
	Strand displacement	5.93
	Meiotic cell cycle	5.91
	Microtubule-based process	5.38

	DNA duplex unwinding	3.99
	Reciprocal meiotic recombination	3.71
	DNA metabolic process	3.07
	Positive regulation of mitotic cell cycle	2.65
	Regulation of cell cycle G2/M phase transition	2.59
	Regulation of cell division	2.38
	Blastocyst growth	2.26
	Cell cycle G1/S phase transition	2.22
	Establishment of chromosome localization	2.18
	Regulation of DNA recombination	2.17
	Response to radiatin	1.90
	Cytokinesis	1.87
	Cellular process	1.84
	Telomere organization	1.47
	Telomere organization	1.45
	Kinetochore assembly	1.33
GC cluster 5 (n = 123), SD 3E	None	
Highly PGC specific (n = 89), SD 8A	Regulation of gene expression, epigenetic	1.83
	Regulation of gene expression, epigenetic	1.71
Female specific (n = 69), SD 11B	None	
Male specific (n = 15), SD 11D	None	
hPGC-specific (n = 119), SD 10D	Meiotic cell cycle	2.41
	DNA alkylation	1.75
	Nucleic acid metabolic process	1.45
PGCLC-specific (n = 82), SD 10B	None	
GC-genes in many (17 or more) tumors (n = 138), SD 4B	Cell cycle	14.83
	Chromosome segregation	4.91
	DNA biosynthetic process	4.41
	DNA synthesis involved in DNA repair	3.07
	Microtubule-based process	2.87
	Centromere complex assembly	2.66
	Response to radiation	2.37
	Regulation of centrosome cycle	2.23
	DNA metabolic process	2.01
	Mitotic DNA replication	1.96
	Positive regulation of mitotic cell cycle	1.88
	Positive regulation of mitotic cell cycle	1.86
	Mitotic cytokinesis	1.40
TGCT-only (n = 83), SD 5B	Nucleic acid metabolic process	5.52
	Multi-organism reproductive process	3.12
	DNA methylation or demethylation	3.00
	Meiosis I	1.67
	Single organism reproductive process	1.62
	Stem cell population maintenance	1.45
Validated on protein-level (n = 201), SD 12	Chromosome segregation	9.86
	DNA replication	4.23
	Cell cycle checkpoint	3.80
	Double-strand break repair	3.14
	Microtubule-based process	2.39
	Mitotic sister chromatid segregation	2.22

	DNA-dependent DNA replication	2.18
	DNA synthesis involved in DNA repair	2.07
	Meiotic nuclear division	1.60
	Histone H3-K9 methylation	1.41

Enrichment equals -$\log_{10}$(p), where 1.3 is equivalent to p = 0.05 and p represents the geometric mean of p-values in an annotation cluster. Only a description of the first term of each statistically significant (enrichment > 1.3) annotation cluster is shown. Full results are shown in corresponding supplementary data (SD) for each subset.

Table S2. GC-genes that are expressed in PGCs and fall into multiple subgroups of interest for further evaluation.

Gene ID	Cell surface	Highly specific to the embryonic germline	Validated on the protein level *
APOBEC3B		X	CPTAC
BFSP2		X	HPA
CD3EAP		X	CPTAC
CHST4		X	CPTAC
CNGA1		X	CPTAC
CRYBB1		X	HPA
CST1		X	CPTAC
FAM111B		X	CPTAC
HAPLN1		X	CPTAC
HIST1H1B		X	CPTAC
HMMR	X		CPTAC
HYAL4	X	X	
KCNH5	X		CPTAC
NANOG		X	HPA
NAT1	X	X	CPTAC
POLR2J3		X	CPTAC
SPA17	X		CPTAC
SPC24		X	CPTAC
TIMM8A		X	CPTAC
UHRF1		X	CPTAC
ULBP3	X		CPTAC
WNT7A	X		HPA + CPTAC

* proteins are validated on the protein level if they are either validated through HPA data (i.e. not expressed in any normal somatic tissue and expressed in at least one tumor type) or CPTAC data (i.e. higher expression in tumor samples compared to normal tumor tissues).
