# Supplementary material for: Tumors Widely Express Hundreds of Embryonic Germline Genes
Source: Cancers (Basel). 2020 Dec 17;12(12):3812. doi: 10.3390/cancers12123812 (PMC7766889; doi:10.3390/cancers12123812)

Supplementary figure 1. Selection of genes based on the expression in three datasets. Because we compare gene expression levels from multiple sources with distinct distributions, we cannot simply compare their values (x-axis). Thus, we determined a cut-off for each dataset to in- or exclude genes.

a/**b**. For gene expression in human primordial germ cells, genes with a maximum gene expression in either female or male hPGCs <0.72 (**a**) and <0.50 in PGCLCs (**b**) were considered background noise and were excluded.
c. Likewise, in order to only include genes that are exclusive to (primordial) germ cells and cancer, genes with an expression >3.0 in any normal somatic tissue were also excluded.
d. Finally, we selected for genes with an expression >2.3 in at least one of 33 tumor types.

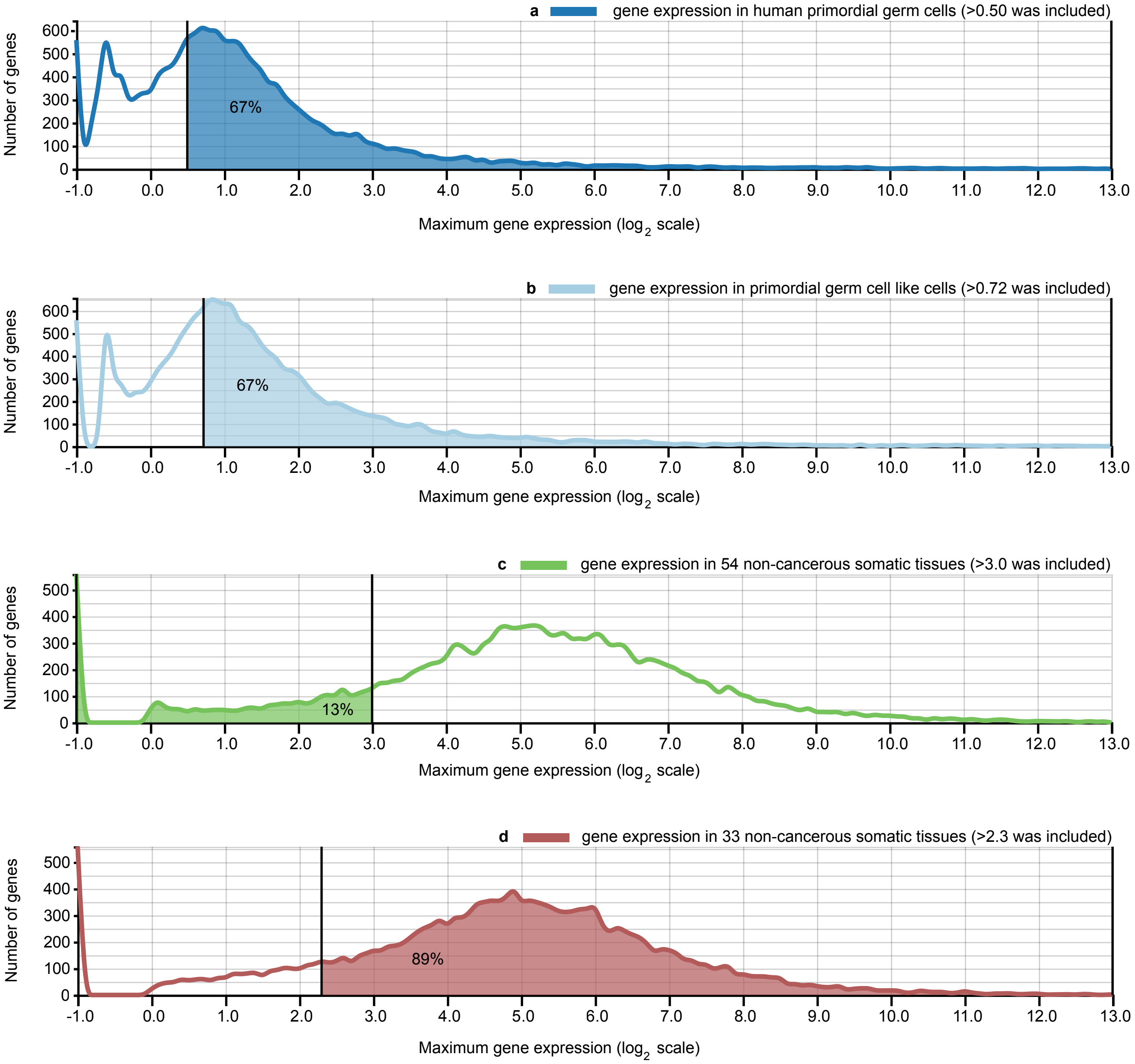

Supplement: Supplementary file 1 [file cancers-12-03812-s001.zip › cancers-1033232-XML suppl/Supplementary figure 1.pdf]
