# Supplementary material for: Tumors Widely Express Hundreds of Embryonic Germline Genes
Source: Cancers (Basel). 2020 Dec 17;12(12):3812. doi: 10.3390/cancers12123812 (PMC7766889; doi:10.3390/cancers12123812)

Supplementary figure 2. Enrichment plot of gene set enrichment analysis (GSEA) of various gene sets

a. All embryonic GC-genes (n = 672)
b. Embryonic GC-gene cluster 1 (n = 105)
c. Embryonic GC-gene cluster 2 (n = 167)
d. Embryonic GC-gene cluster 3 (n = 97)
e. Embryonic GC-gene cluster 4 (n = 180)
f. Embryonic GC-gene cluster 5 (n = 123)

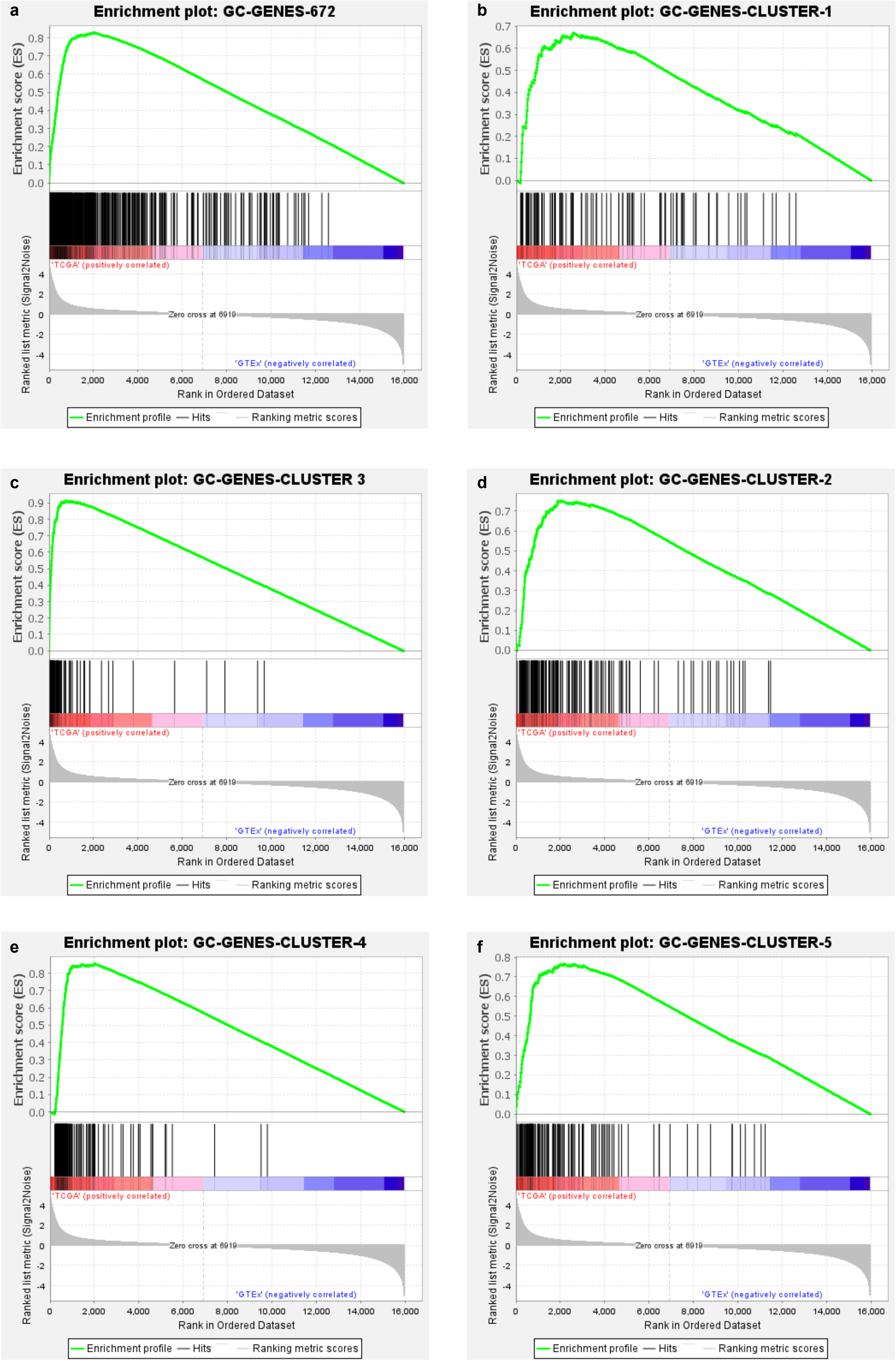

Supplement: Supplementary file 1 [file cancers-12-03812-s001.zip › cancers-1033232-XML suppl/Supplementary figure 2.pdf]
